# Macroscopic Neural Oscillation during Skilled Reaching Movements in Humans

**DOI:** 10.1155/2016/2714052

**Published:** 2016-07-25

**Authors:** Hong Gi Yeom, June Sic Kim, Chun Kee Chung

**Affiliations:** ^1^Interdisciplinary Program in Neuroscience, Seoul National University, Seoul 151-742, Republic of Korea; ^2^Department of Brain and Cognitive Sciences, College of Natural Sciences, Seoul National University, Seoul 151-742, Republic of Korea; ^3^Department of Neurosurgery, Seoul National University Hospital, Seoul 110-744, Republic of Korea

## Abstract

The neural mechanism of skilled movements, such as reaching, has been considered to differ from that of rhythmic movement such as locomotion. It is generally thought that skilled movements are consciously controlled by the brain, while rhythmic movements are usually controlled autonomously by the spinal cord and brain stem. However, several studies in recent decades have suggested that neural networks in the spinal cord may also be involved in the generation of skilled movements. Moreover, a recent study revealed that neural activities in the motor cortex exhibit rhythmic oscillations corresponding to movement frequency during reaching movements as rhythmic movements. However, whether the oscillations are generated in the spinal cord or the cortical circuit in the motor cortex causes the oscillations is unclear. If the spinal cord is involved in the skilled movements, then similar rhythmic oscillations with time delays should be found in macroscopic neural activity. We measured whole-brain MEG signals during reaching. The MEG signals were analyzed using a dynamical analysis method. We found that rhythmic oscillations with time delays occur in all subjects during reaching movements. The results suggest that the corticospinal system is involved in the generation and control of the skilled movements as rhythmic movements.

## 1. Introduction

The neural mechanism of skilled movements has been considered to differ from that of rhythmic movement [1]. Skilled movements, such as reaching and grasping, are nonperiodic and are consciously controlled by the brain, while rhythmic movements such as locomotion are repetitive and stereotypical. Although rhythmic movements can be controlled voluntarily, these movements are usually controlled autonomously by the spinal cord and brain stem. A central pattern generator (CPG) in the spinal cord produces periodic oscillatory patterns [1, 2]. The CPG has been considered to be associated with the control of rhythmic movement [3–5].

However, it has been suggested that not only the cortical circuit but also the neural networks in the spinal cord may be involved in skilled movements [6–12]. Moreover, Rokni and Sompolinsky demonstrated that various natural movements can be generated by the linear summation of simple oscillatory components [13]. When considering Fourier theory, it is reasonable to presume that all of the complicated signals can be approximated by the linear summation of sine and cosine signals [14]. The suggestion of generating the various movements from simple components also corresponds to the perspective of dynamic systems, suggesting that most neural activity in the motor cortex will be internal processes that drive desired movements [15]. A recent study reported an important phenomenon. The study, based on neural dynamical analysis, demonstrated that rhythmic oscillations corresponding to the movement frequency also occur during skilled reaching movements [16] as rhythmic movements [16–19]. This implies that diverse skilled movements can be generated via CPG, similar to the neural mechanism of rhythmic movements.

However, in the previous study, a very small motor area in a monkey was measured at a microscopic level. Therefore, whether the rhythmic oscillations are generated in CPG or the cortical circuit in the motor cortex causes the oscillations is unclear. Moreover, the occurrence of the rhythmic oscillations corresponding to the movement frequency has not been confirmed in humans.

Various pathways connect the cortex and spinal cord. The direct corticomotoneuronal (CM) pathway connects the motor cortex to spinal motoneurons. Indirect pathways might connect other sensorimotor cortices, such as the premotor (PM), supplementary (SMA), cingulate (CMA), and primary somatosensory (S1) areas, to the spinal cord [20]. Therefore, if the rhythmic oscillations occur from the spinal cord and are delivered to the broad motor-related cortex, similar rhythmic oscillations with time delays should be found in macroscopic neural activity (Figures 3(a) and 3(b)). Here, we examine whether similar rhythmic oscillations with time delays are exhibited in macroscopic neural activity during reaching movements in humans. To investigate neural activity, we measured whole-brain MEG signals during reaching movements. We analyze the MEG signals using an analysis method, j principle component analysis (jPCA), where j implies an imaginary part in a complex conjugate. The method reveals the dynamical characteristics of the neural activity [16]. If there are similar oscillatory patterns with time delay, the projections of the oscillations onto the jPC planes will be rotated. Therefore, we can easily investigate whether similar rhythmic oscillations occur or not by examining the projections. Moreover, the results will indicate whether the rhythmic oscillations occur from spinal cords or not.

## 2. Materials and Methods

### 2.1. Experiment and Data Acquisition

**Figure 1:**
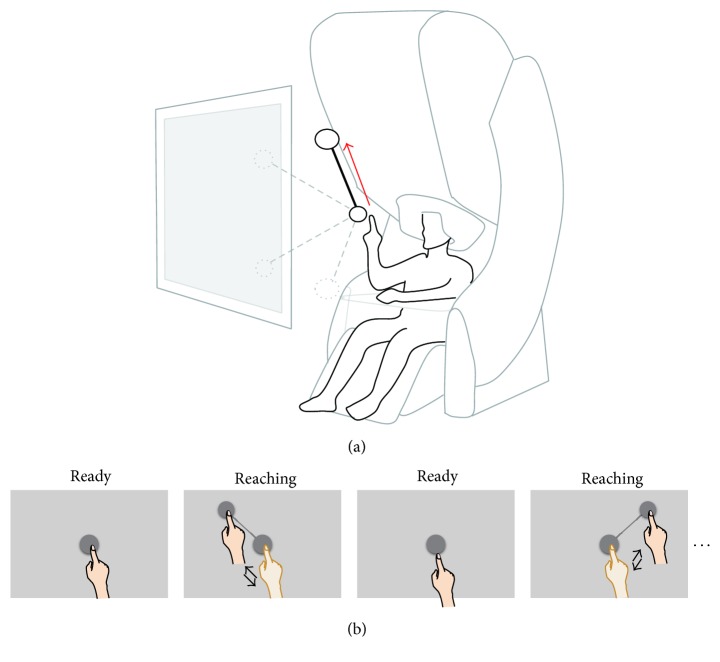
Experiment paradigm. (a) Illustration of the virtual visual stimuli and MEG acquisition system. MEG signals and hand positions were recorded simultaneously during reaching movements. Subjects were instructed to perform a center-out reaching task according to stereographic images on a screen. The target sphere appeared randomly on one of the four corners (upper-left, upper-right, bottom-left, and bottom-right). The red arrow illustrates the example of the reaching movements when the target is shown at the upper-left. (b) Drawings showing the sequence of visual stimuli and instructed behaviors. At the beginning of the experiments, a sphere was shown on the center of the screen. After 4 s, the target was presented for 1 s on one of the four corners. During this time, the subjects were instructed to shift their index finger from the center to the target and return to the center according to the connecting line by moving their right arm as fast as possible. This sequence was repeated during the experiments.

Nine healthy subjects (age: 19–37 years; five males and four females) participated in the experiment. All subjects were right-handed (Edinburgh Handedness Inventory scores were above 80). A 306-channel whole-head MEG system (VectorView*™*, Elekta Neuromag Oy, Helsinki, Finland) was used to measure neural activity during reaching movements (Figure 1(a)). The MEG system has 306 sensors grouped in triplets consisting of 2 planar gradiometers and 1 magnetometer distributed at 102 locations. To record arm position, a three-axis accelerometer (KXM52, Kionix, NY, USA) was attached to the index finger of the right hand using the Velcro band. The accelerometer signals were recorded simultaneously with the MEG signals. The sampling frequency of the MEG and the accelerometer signals was 600.615 Hz. The experiment was approved by the Institutional Review Board of the Seoul National University Hospital (IRB number 1105-095-363). Subjects were instructed to perform a center-out reaching task according to stereographic images on a screen. To minimize movement artifacts, a cushion was placed under the subject's elbow during the experiment.

At the beginning of the experiments, a sphere was shown on the center of the screen for 4 s and a target sphere with a stick connecting it to the center sphere was presented on one corner for 1 s. The target sphere appeared randomly on one of the four corners (upper-left, upper-right, bottom-left, and bottom-right). During this time, the subjects were instructed to shift their index finger from the center to the target and return to the center according to the connecting line by moving their right arm as fast as possible (Figure 1(b)). This sequence was repeated during the experiments. For each subject, 60 trials were measured for each direction. The distances from the center to the target were ~20 cm. Because we used stereographic images to represent the target, the distance measurement was not accurate. The distances from the center to the target were identical in all subjects. Because the directions of the target were randomly presented, we did not consider the variation of the intertrial interval for habituation effects. Although the reaction times were slightly different (see Section 3), there is no strange trial. Further details are also described elsewhere [21, 22].

### 2.2. Signal Preprocessing

**Figure 2:**
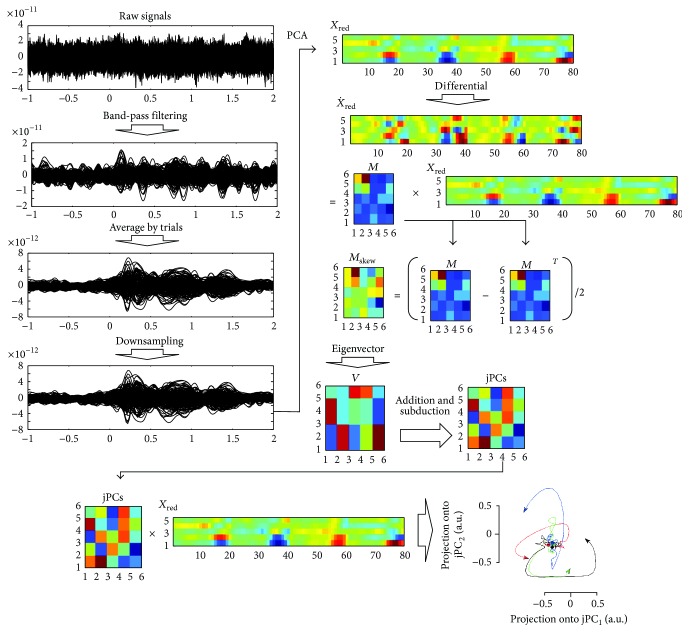
Signal process of jPCA analysis. The segmented MEG signals from 1-2 s before the cue onset were band-pass filtered between 0.5 and 8 Hz. After filtering, the MEG signals were averaged by trials. The averaged MEG signals were downsampled at 20 ms. Matrices* M* and *M*_skew_ were calculated from downsampled signals. The eigenvectors of *M*_skew_ produced jPCs. Projections on the jPC plane were illustrated by multiplying jPCs and *X*_red_.

The MEG equipment measures the changes of a magnetic field. Therefore, it is influenced by external signals, such as line noise, and biological artifacts, such as cardiac and muscle activity. To reduce the noise in the MEG signals, the spatiotemporal signal space separation (tSSS) method was applied [23]. The tSSS separates external interference signals of the brain by spatiotemporal methods and eliminates the interference. All data processing was performed using MATLAB 2008b (Mathworks, Natick, MA, USA). We used 204 gradiometer signals among the 306 channels for data analysis because the characteristics of the gradiometer and magnetometer sensors differ and the signal-to-noise ratio (SNR) of the gradiometers is better than that of the magnetometers [24]. The MEG signals were band-pass filtered between 0.5 and 8 Hz. The filtering band was determined by time-frequency analysis based on our previous study [21]. The MEG signals of the frequency band represent the characteristics of reaching movements [21]. To minimize artifacts, independent component analysis (ICA) was applied, which is implemented in EEGLAB [25]. Artifacts such as EOG were removed by eliminating the artifact components. The signals were segmented from −1 to 2 s after the cue onset. After the segmentation, the MEG signals were averaged by trials. The averaged MEG signals were downsampled to 50 Hz. jPCA was applied to the preprocessed signals. The process of the analysis is explained in the next section and in Figure 2.

### 2.3. Dynamical Analysis jPCA

Neural networks in the brain consist of billions of neurons. Although the activities of some neurons in the motor cortex will reflect movement parameters, most neural activities will be an internal process to generate the motor commands [15]. Therefore, the signals are difficult to analyze because the patterns of neural activity are various and complex. To investigate the neural process, a dynamical analysis method, jPCA, was proposed [16]. jPCA describes the dynamical relationship between current and subsequent neural activities on a low-dimensional plane. It represents the dynamic relationship with rotation according to the time flow. If there are consistent phase-differences between neural activities, the projections on the jPC planes will be rotated; however, if there is no consistent change, meaningless small movements will be shown. Moreover, if the sign of the phase-difference changes, the projections will be rotated in the opposite direction. This reveals the change in the dynamic relationship between the current and subsequent neural activities. jPCA is an intuitive method of analyzing the dynamic characteristics of neural activities. jPCA finds informative planes and projects the neural data onto the planes. The projected neural data represents the rotational structure in the data.

The relationship between the current and next neural activities can be expressed as follows:

(1) $$\begin{array}{c} \dot{X}=MX, \end{array}$$

where *X* is a matrix of size *n* × *ct* describing the neural activities. The preprocessed MEG signals as described in Section 2.2 were applied to jPCA as *X*. *n* is the number of MEG channels (*n* = 204). *c* is the number of conditions (*c* = 4), and *t* is the number of time points (*t* = 151). $\dot{X}$ is a derivative of *X* and *M* represents the relationship between the neural activity and its derivative.

jPCA applies the traditional principal component analysis (PCA) to reduce the dimensionality of *X* from *n* × *ct* to *k* × *ct* (*k* = 6). The reduced neural activity will be expressed as *X*_red_. Equation (1) can be represented as follows:

(2) $$\begin{array}{c} {\dot{X}}_{red}=MX_{red}. \end{array}$$

*M* can be calculated by linear regression. *M* is a combination of symmetric transformation and skew transformation. *M* can be divided as follows:

(3) $$\begin{array}{c} M=M_{symm}+M_{skew}, \end{array}$$

where *M*_symm_ and *M*_skew_ are defined as *M*_symm_ = (*M* + *M* ^*T*^)/2 and *M*_skew_ = (*M* − *M* ^*T*^)/2.

Equation (2) can be expressed as follows:

(4) $$\begin{array}{c} {\dot{X}}_{red}=M_{skew}X_{red}. \end{array}$$

Because *M*_skew_ has imaginary eigenvalues, it captures the rotational dynamics of neural activity. To express complex eigenvectors *V*_1_ and *V*_2_ of *M*_skew_ on a real plane, the jPC can be defined as jPC_1_ = *V*_1_ + *V*_2_ and jPC_2_ = j(*V*_1_ − *V*_2_).

The projection onto the jPC can be calculated as *X*_jPCA_ = (jPC_1_; jPC_2_) × *X*_red_. Further details are provided in [16].

**Figure 3:**
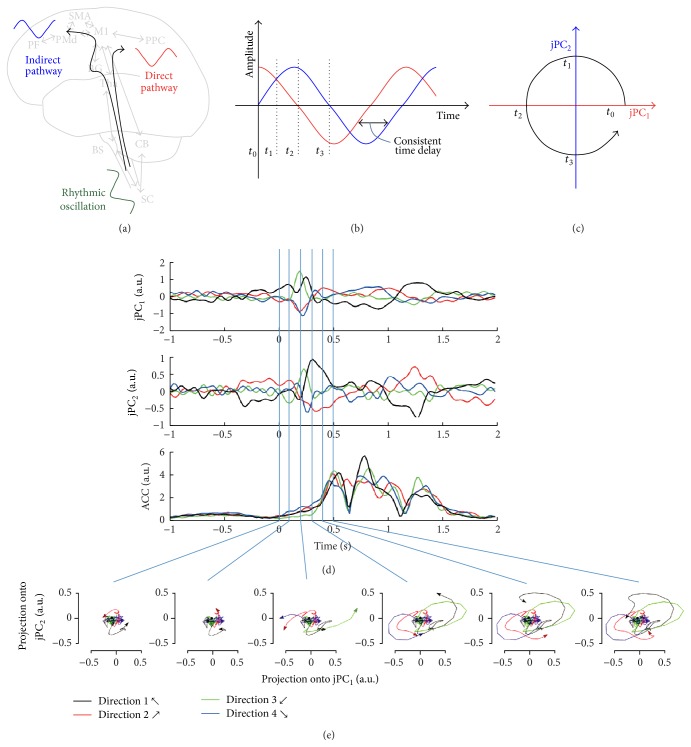
Illustration of a simple rhythmic oscillation model to draw a circle on the jPC planes and results of jPCA analysis. (a) Delivery of a rhythmic oscillation to the different area through direct and indirect pathways with different time delays. (b) Oscillations of the different areas with similar patterns and a consistent time delay. (c) Rotation of the oscillations on the jPC planes. (d) jPCs and root-mean squares (RMSs) of accelerometer signals with time. The different-colored signals represent the movements in different directions. 0 ms indicates the stimulus onset time. (e) Projections on the jPC planes are shown at 0, 100, 200, 300, 400, and 500 ms. The projections were rotated prior to movement onset. The results suggest that the oscillations are related not only to movement execution but also to movement preparation.

**Figure 4:**
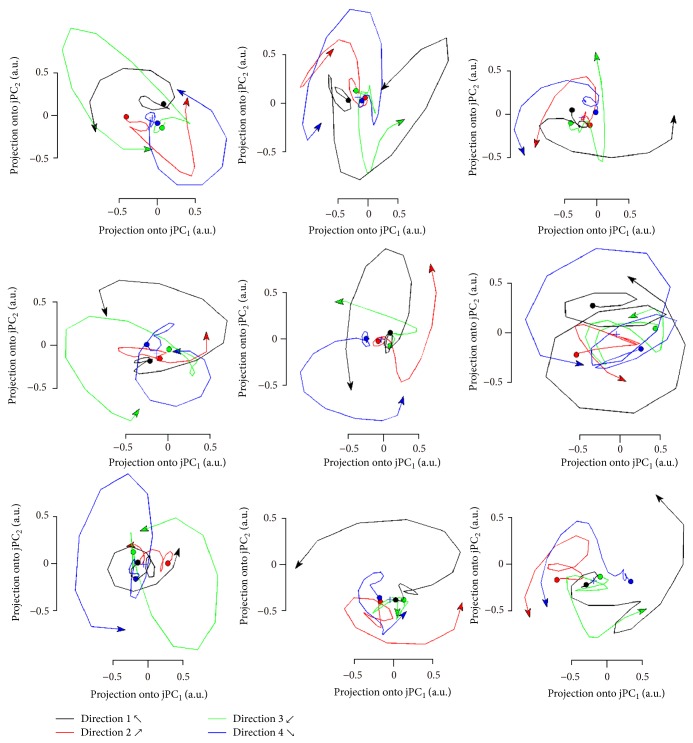
Projections on the jPC planes for all subjects from −100 to 300 ms after presentation of visual stimuli. Each subfigure illustrates the projections from each subject. The different-colored signals represent the movements in different directions. The projection of the oscillation rotated to the same direction (counterclockwise) for all conditions (reaching of different direction) for all subjects. This suggests that the same neural dynamics are involved in reaching movements irrespective of reaching direction.

Our results show the projections on one on each jPC (Figure 3(d)) or on the two-dimensional space of jPC_1_ and jPC_2_ (Figure 4).

## 3. Results

Figure 3 shows a simple rhythmic oscillation model that draws a circle on jPC planes and results from jPCA. To draw a circle on jPC planes as in Figure 3(c), the rhythmic oscillations of jPC_1_ and jPC_2_ should have a similar pattern. Moreover, the time difference between oscillations should be consistent as in Figure 3(b). Figure 3(d) illustrates jPCs and root-mean square (RMS) of the accelerometer signals over time. Projections on jPC planes are also shown at 0, 100, 200, 300, 400, and 500 ms in Figure 3(e). The different colors of the signals represent the movements of different directions. 0 ms indicates the stimulus onset time. The average movement onset was 316 ± 58 ms (mean ± standard deviation) from stimulus onset. In contrast, the rhythmic oscillations began at 133 ± 69 ms. We compared the onset times between the movements and the rhythmic oscillations using a paired* t*-test. The onsets differed significantly (*p* < 0.001) between the movements and the rhythmic oscillations. After the presentation of visual stimuli, the rhythmic oscillations of jPCs occurred before arm movements, as shown in Figure 3(d). Projections on jPC planes show clearer results. Before movement onset, the projections of the neural oscillations were rotated.

Figure 4 shows the projections on jPC planes for each subject from −100 to 300 ms after presentation of visual stimuli. The duration corresponds to the time taken for movement preparation. The projection of the oscillation rotated in the same direction (counterclockwise) for all conditions (reaching different directions) for all subjects. To rotate in the same direction for different conditions, the relation between jPC_1_ and jPC_2_ should be invariant irrespective of the movement direction.

Table 1 shows a summary of jPCA. The second row describes the data variance captured by jPC plane. The mean of the data variance was 0.235 ± 0.059. The third row is fit quality provided by *M*_skew_, in the first two jPCs. The mean of the fit quality from two jPCs was 0.235 ± 0.075.

## 4. Discussion

In this study, we demonstrated that the neural mechanisms of rhythmic movements and skilled movements are similar. We showed that the corticospinal system is involved in the generation of skilled movements by means of a dynamic analysis of macroscopic neural data. Although our result does not concur with previous knowledge, it supports the suggestion made in recent studies that the spinal cord mediates skilled movements [6–12].

### 4.1. Neural Mechanism of Skilled Movements

It is unclear whether the rhythmic oscillations are generated in the spinal cord or in the motor cortex. We hypothesize that if the rhythmic oscillations are derived by the corticospinal system, oscillations with similar pattern and consistent time delay should be found in macroscopic neural activity (Figures 3(a) and 3(b)). To investigate this hypothesis, we measured and analyzed MEG signals during reaching movements in humans. The results showed that projections on the jPC planes rotated in all subjects (Figure 4). This means that the major components of the neural activity have a similar pattern and consistent time delay. Therefore, it implies the possibility that skilled movements are generated by the corticospinal system. Moreover, the projection of the oscillation rotated in the same direction for all conditions. This suggests that the same neural dynamics are involved in reaching movements, irrespective of reaching direction.

Our perspective corresponds to the suggestion of a common intrinsic structure controlling the reaching movements [16, 26]. When we consider that the cortex also contributes to controlling rhythmic movements [7, 27], the neural mechanisms of rhythmic movements and skilled movements are similar.

Despite numerous studies of a CPG in animals, almost all studies in humans used indirect results [28]. We also did not measure spinal cord activity directly. Therefore, we should be cautious when interpreting the results. Nevertheless, our results provide evidence that the corticospinal system is involved in skilled movement, such as rhythmic movement.

### 4.2. Neural Oscillations Involved in Movement Generations

Iteration of descending motor commands and ascending sensory feedback could be represented by rhythmic patterns. Therefore, the rhythmic oscillations could be considered products generated by sensory feedback, such as kinematic parameter or visual feedback [28–32]. However, our results show that the rhythmic oscillation occurred prior to movement onset (Figures 3(d), 3(e), and 4). Because sensory feedback could not generate the oscillations before the movements, it implies that there is another mechanism of generating the rhythmic oscillations. It has been suggested that neural networks in the spinal cord may also be involved in skilled movements. We hypothesized that if the rhythmic oscillations are made by CPG and delivered to the broad motor-related area through direct and indirect pathways, similar patterns of time delay will be observed at a macroscopic level. We verified our hypothesis using the dynamical analysis method. Therefore, our results suggest that the spinal cord could be involved in the movement generation.

When a subject is aware of a target position in a motor planning task, the reaction time required to reach the target after the go cue was about 240 ms [33]. Because the visual processing requires less than 150 ms [34], and the execution-related time will be about 90 ms. In our experiment, the movement onset time (316 ms) may include the time for visual processing, motor planning, and execution. Therefore, the difference (76 ms) between 316 ms and 240 ms is related to motor planning. Thus, 166 ms (76 + 90 ms) may be required for movement planning and execution. The rhythmic oscillation occurred 183 ms (316 − 133 ms) prior to movement onset, suggesting that the rhythmic oscillations are related to movement generation.

## 5. Conclusions

We showed that neural oscillations occur at a macroscopic level in humans during skilled movements. It seems that a common intrinsic structure generates the oscillations, irrespective of movement direction. The intrinsic structure is involved in not only movement execution but also movement generation. The neural oscillations could be generated in the spinal cord and the oscillations might influence movements by means of corticospinal interaction. This implies that the neural mechanism of skilled movements might be similar to that of rhythmic movements.

## Figures and Tables

**Table 1:** Summary of jPCA. Captured variance, data variance captured by the jPC plane; fit by 2 jPCs, fit quality provided by *M*_skew_ in the first two jPCs.

	Sub 1	Sub 2	Sub 3	Sub 4	Sub 5	Sub 6	Sub 7	Sub 8	Sub 9	Mean ± standard deviation
Captured variance	0.226	0.382	0.180	0.212	0.190	0.231	0.242	0.243	0.212	0.235 ± 0.059
Fit by 2 jPCs	0.164	0.226	0.157	0.168	0.245	0.250	0.237	0.401	0.271	0.235 ± 0.075
